# Cardiac Remodeling in Heart Failure: Role of Pyroptosis and Its Therapeutic Implications

**DOI:** 10.3389/fcvm.2022.870924

**Published:** 2022-04-18

**Authors:** Ruoning Chai, Wenjing Xue, Shuqing Shi, Yu Zhou, Yihang Du, Yuan Li, Qingqiao Song, Huaqin Wu, Yuanhui Hu

**Affiliations:** ^1^Department of Cardiovascular, Guang'anmen Hospital, China Academy of Chinese Medical Sciences, Beijing, China; ^2^Department of Clinical Medicine, Beijing University of Chinese Medicine, Beijing, China; ^3^Department of Internal Medicine, Guang'anmen Hospital, China Academy of Chinese Medical Sciences, Beijing, China; ^4^Department of Clinical Medicine, Shaanxi University of Chinese Medicine, Xianyang, China

**Keywords:** pyroptosis, cardiac remodeling, cardiac fibrosis, heart failure, inflammation

## Abstract

Pyroptosis is a kind of programmed cell death closely related to inflammation. The pathways that mediate pyroptosis can be divided into the Caspase-1-dependent canonical pathway and the Caspase4/5/11-dependent non-canonical pathway. The most significant difference from other cell death is that pyroptosis rapidly causes rupture of the plasma membrane, cell expansion, dissolution and rupture of the cell membrane, the release of cell contents and a large number of inflammatory factors, and send pro-inflammatory signals to adjacent cells, recruit inflammatory cells and induce inflammatory responses. Cardiac remodeling is the basic mechanism of heart failure (HF) and the core of pathophysiological research on the underlying mechanism. A large number of studies have shown that pyroptosis can cause cardiac fibrosis, cardiac hypertrophy, cardiomyocytes death, myocardial dysfunction, excessive inflammation, and cardiac remodeling. Therefore, targeting pyroptosis has a good prospect in improving cardiac remodeling in HF. In this review, the basic molecular mechanism of pyroptosis is summarized, the relationship between pyroptosis and cardiac remodeling in HF is analyzed in-depth, and the potential therapy of targeting pyroptosis to improve adverse cardiac remodeling in HF is discussed, providing some ideas for improving the study of adverse cardiac remodeling in HF.

## Introduction

Heart failure (HF) is a clinical syndrome caused by insufficient heart pumping function and is the terminal stage of cardiovascular diseases, affecting approximately 64.3 million people worldwide (1, 2). In developed countries, the prevalence of known HF is generally estimated at 1% to 2% of the general adult population (3, 4), the study indicated patients with HF had 87%, 73%, 57%, and 35% survival at 1, 2, 5, and 10 years (5), and HF events increased the risk of death by five times. The absolute number of HF patients will continue to increase due to population aging, global population growth, and improved survival rates after diagnosis, as well as a gradual increase in younger patients (<55 years). North America had the highest hospitalization rate for HF (11/100 person-years) (6), HF remains a serious clinical and public health problem (7–10). Despite significant advances in drug and instrumental treatment of HF over the past two decades, patient outcomes and quality of life remain inadequate, which may be closely related to a focus on symptomatic treatment and a lack of in-depth discussion of pathophysiology (9).

Left ventricular function is an important indicator for evaluating HF and is also a key point in the study of potential mechanisms in pathophysiology (3). The occurrence and progression of HF are closely related to cardiac remodeling, which is characterized by changes in cardiac structure, shape, and function, and cardiomyocytes death is a key step in cardiac remodeling (11, 12). Cell death is the final stage of cell life, including programmed cell death and non-programmed cell death, of which pyroptosis is a type of programmed cell death that accompanies an inflammatory response (13). Pyroptosis is a double-sided sword, on the one hand, moderate pyroptosis can contribute to cellular homeostasis, effectively prevent excessive cell proliferation and protect the host, on the other hand, excessive pyroptosis can cause cardiac fibrosis, myocardial hypertrophy, cardiomyocytes death, myocardial dysfunction, excessive inflammation and promote cardiac remodeling (14). The most significant difference between pyroptosis and other modes of cell death lies in the rapid rupture of the plasma membrane, cell expansion, dissolution and rupture of the cell membrane, the release of cell contents and a large number of inflammatory factors, send pro-inflammatory signals to adjacent cells, recruit inflammatory cells and induce inflammatory responses (14–16). Nowadays, many studies have proved that pyroptosis may be an endogenous regulatory factor of cardiovascular diseases and play an important role in cardiovascular diseases (17).

In this review, the basic molecular mechanism of pyroptosis is summarized, the relationship between pyroptosis and cardiac remodeling HF is analyzed in-depth, and the potential therapy of inhibiting pyroptosis to improve cardiac remodeling HF is discussed, which provides some ideas for improving adverse cardiac remodeling HF from the perspective of pyroptosis.

## Overview of Pyroptosis

Pyroptosis, also known as inflammatory necrosis of cells (18), was first observed by Cookson and Brennan in salmonella-infected macrophages and named pyroptosis (19). When pathogens invade a host cell, pattern recognition receptors (PRRs) are capable of recognizing pathogen-associated molecular patterns (PAMPs) and damage-associated molecules patterns (DAMPs) intracellularly, bind to specific ligands, and combine with other proteins to form inflammasome (15, 20, 21). Meanwhile, the canonical pathway also detects cytoplasmic disturbances, recently coined as homeostasis altering molecular properties (HAMPs), and the recognition of HAMPs is through the detection of molecular processes that perturbs cytoplasmic homeostasis (22, 23). PRRs involved in pyroptosis mainly include Nod-like receptors (NLRs) family—NLRP3, NLRP1, NLRP6, NLRP9, NLRC4, PYHIN200 family—Absent in melanoma 2 (AIM2) and TRIM family—Pyrin (24–26). NLRP3 is currently the most famous inflammasome and the noncanonical pathway crosstalks with the canonical pathway *via* NLRP3 (27). NLRs generally contain three domains: an N-terminal adaptor domain [such as CARD or pyrin domain (PYD)], a central nucleotide-binding domain (NBD), and a C-terminal leucine-rich repeat (LRR) domain. NLRP1 contains two additional domains at its C-terminus, a CARD followed by a function to find domain (FIIND) domain. The LRR domains are used to detect bacterial components, the NBD domain is critical for oligomerization and activation, the N-terminal domain is responsible for CARD recruitment and CARD-CARD interactions as well as for activating Caspase-1. As the activation platform of Caspase, the inflammasome plays an important role in the occurrence of pyroptosis. When cells are subjected to different stimuli, the induced pyroptosis pathway is different, which can be divided into the Caspase-1-dependent canonical pathway and the Caspase4/5/11-dependent non-canonical pathway (28, 29).

### The Canonical Pathway of Pyroptosis

**Figure 1:**
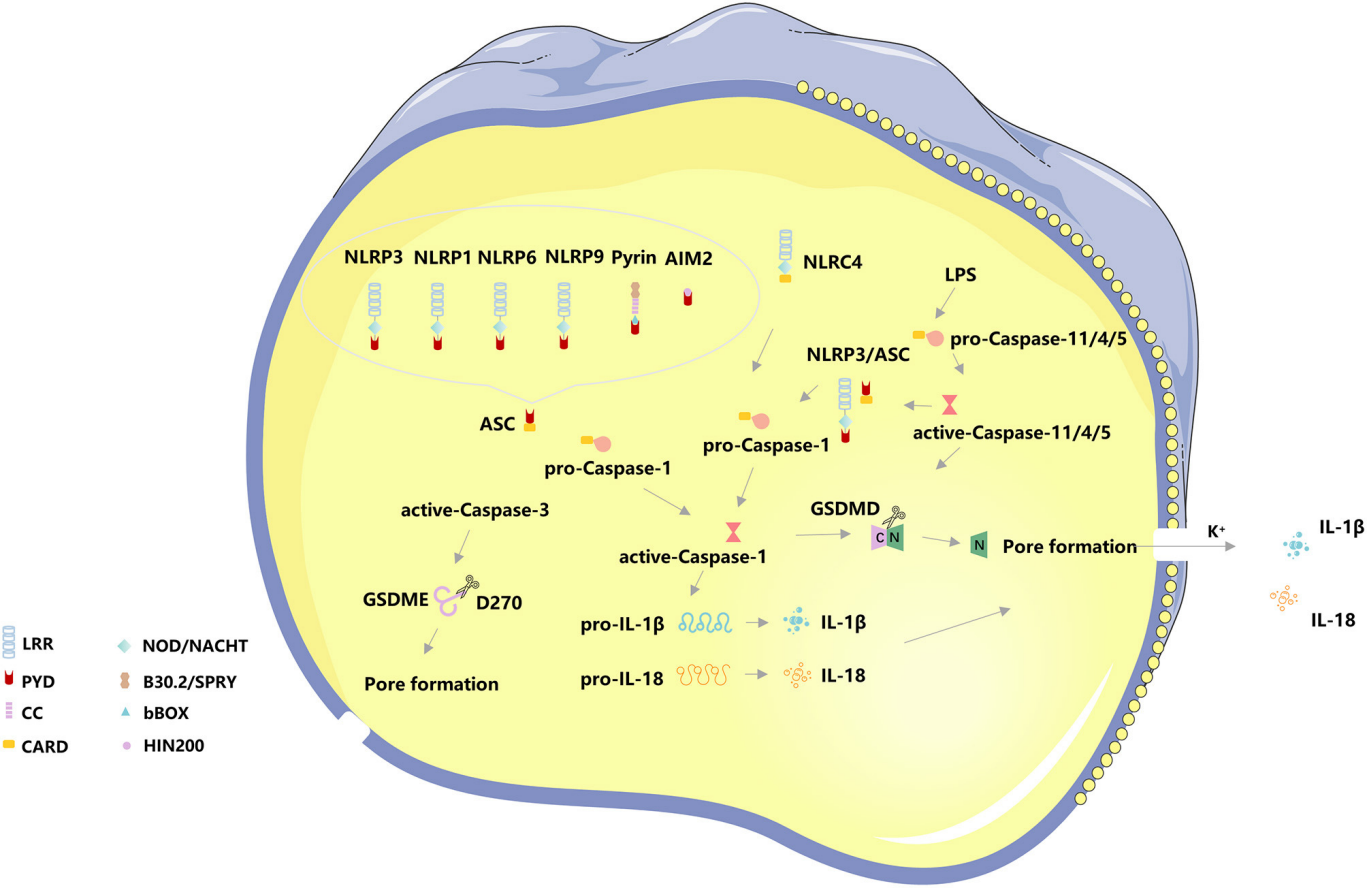
The basic molecular mechanism of pyroptosis. The canonical pathway of pyroptosis, Nod-like receptors protein-3 (NLRP3), NLRP1, NLRP6, NLRP9, absent in melanoma 2 (AIM2), and Pyrin binds to the N-terminal PYD region of the apoptosis-associated speck-like protein (ASC) to activate ASC proteins through protein-protein interactions. The C-terminal CARD domain of ASC and the N-terminal CARD domain of pro-Caspase-1 combine to recruit active-Caspase-1. The binding complex of PRRs, ASC, and pro-Caspase 1 is termed the inflammasome. On the one hand, Caspase-1 recognizes pro-IL-1β and pro-IL-18, converts them into IL-1β and IL-18, and releases them extracellular to expand the inflammatory response, on the other hand, Caspase-1 shear Gasdermin family protein GSDMD to separate its N- and C- domains, N-terminal fragments are released to the membrane, mediating the formation of cell membrane pores, releasing inflammatory factors and inducing pyroptosis. NLRC4 can directly interact with pro-Caspase-1 *via* CARD-CARD to form active-Caspase-1 and induce pyroptosis. The non-canonical pathway of pyroptosis, Caspase4/5/11 can directly bind to lipopolysaccharide (LPS) in the cytoplasm and initiate pyroptosis following cleavage of GSDMD-induced membrane pore formation and subsequent cell membrane rupture. The K^+^ efflux caused by cell membrane pore formation induces activation of the NLRP3/ASC/ Caspase-1 pathway. In addition, Caspase-3 cleaves the Gasdermin family protein GSDME, releasing the N-terminal active fragment to the cell membrane, leading to pyroptosis.

When cells are infected by pathogens or sense endogenous danger signals, PRRs interact with PAMPs or DAMPs and activate the apoptosis-associated speck-like protein (ASC) to activate ASC proteins through protein-protein interactions (21, 30). The C-terminal CARD domain of ASC and the N-terminal CARD domain of pro-Caspase-1 combine to recruit active-Caspase-1 (31). The binding complex of PRRs, ASC, and pro-Caspase 1 is termed the inflammasome. On the one hand, Caspase-1 recognizes pro-IL-1β and pro-IL-18, converts them into IL-1β and IL-18, and releases them extracellular to expand the inflammatory response, on the other hand, Caspase-1 shear Gasdermin family protein GSDMD to separate its N- and C- domains, N-terminal fragments are released to the membrane, mediating the formation of cell membrane pores, releasing inflammatory factors and inducing pyroptosis (32). The canonical pathway also utilizes toll-like receptors (TLRs) for priming certain PRRs to enhance immune responses, TLR4 can upregulate NLRP3 (33, 34) and GSDMD (35) to promote pyroptosis *via* Nek7, GBP5, and NF-κB signaling. NLRC4 can directly interact with pro-Caspase-1 *via* CARD-CARD to form active-Caspase-1 and induce pyroptosis (36, 37) (Figure 1).

### The Non-canonical Pathway of Pyroptosis

In addition to the canonical pathway of pyroptosis, the CARD domain of pro-Caspase 4/5/11 directly interacts with the intracellular lipopolysaccharides (LPS) of Gram-negative bacteria, causing a significant conformational rearrangement of pro-Caspase 4/5/11 (36, 37) and resulting in oligomerization and autoproteolysis. After oligomerization, Caspase 11 auto-proteolyze after D285 with a sequence of MEAD|A to gain full activity in proteolyzing GSDMD (38, 39). However, activated Caspase 4/5/11 has been considered only to recognize GSDMD and cleave at D275 of hGSDMD or D276 of mGSDMD for generating pyroptotic pores (38, 39). Meanwhile, Caspase 4/11 were activated by guanylate-binding proteins 1-4 (38), and Caspase 4/5/11 also crosstalk with NLRP3, both of them can process to pyroptosis. The K^+^ efflux caused by cell membrane pore formation induces activation of the NLRP3/ASC/ Caspase-1 pathway (39, 40). The non-canonical pathway has also been considered to involve mitochondrial dysfunction, such as the release of mitochondrial reactive oxygen species (ROS) and mitochondrial DNA (mtDNA) (39). In addition, Caspase-3 cleaves the Gasdermin family protein GSDME, releasing the N-terminal active fragment to the cell membrane, leading to pyroptosis. The distribution and expression levels of GSDME determine the mode of cell death activated by Caspase-3, and when cells overexpress GSDME, activated Caspase-3 induces pyroptosis (40) (Figure 1).

### The Regulation of NLRP Inflammasome on Pyroptosis

NLRP Inflammasome belongs to the NLRs family which generally contains three domains: an N-terminal adaptor domain [such as CARD or pyrin domain (PYD)], a central nucleotide-binding domain (NBD), and a C-terminal leucine-rich repeat (LRR) domain. NLRP1 contains two additional domains at its C-terminus, a CARD followed by a function to find domain (FIIND) domain. The LRR domains are used to detect bacterial components, the NBD domain is critical for oligomerization and activation, the N-terminal domain is responsible for CARD recruitment and CARD-CARD interactions as well as for activating Caspase-1.

#### NLRP3

The NLRP3 inflammasome is the best studied and possesses the most complex signaling of all inflammasomes. NLRP3 must first be primed by a cytokine receptor or another PRR (41). When NLRP3 is knocked out in mice or antagonized, there is a significant reduction of pyroptosis. Interestingly, lysosomal membrane damage results in K^+^ efflux and the release of lysosomal protease cathepsin B, both of which initiate NLRP3 inflammasome activation (42). The protein NIMA-related kinase 7 (NEK7), which participates by regulating NLRP3 inflammasome formation following potassium release and preventing inflammasome formation during mitotic division stages of the cell cycle, is a crucial player during NLRP3 activation (43, 44). Co-crystal structures show that NEK7 directly binds to two neighboring NLRP3 subunits (44). Knockdown of RBP4 alleviated ischemia-hypoxia-induced activation of NLRP3 inflammasome signaling and pyroptosis in cardiomyocytes (45). After activation, the NLRP3 receptor recruits the ASC by PYD-PYD interaction, assembling the NLRP3 inflammasome. NLRP3 crosstalks with other innate immune pathways (46, 47), indicating that the inhibition of the NLRP3 could have a prospect effect on inflammatory responses. Therefore, NLRP3 is a hopeful drug target for patients with inflammatory diseases (48).

#### NLRP1

NLRP1 was the first discovered NLR family member that was discovered to be involved in the formation of an inflammasome complex. The NLRP1 possesses a C-terminal CARD domain and an N-terminal pyrin domain so that it can activate pro-Caspase 1 without ASC oligomerization, leading to IL-1β secretion (49, 50). It is coded by a single gene in humans with three homologs NLRP1a, b, and c in mice (51). In terms of human disease, the NLRP1 inflammasome is genetically associated with several other autoimmune diseases. In addition, NLRP1 can be activated by muramyl dipeptide and ATP in humans (52). Interestingly, inhibitors of dipeptidyl peptidases 8 and 9 (DPP8/9) can selectively activate NLRP1 and its related protein CARD8 in both mouse and human lymphocytes (53). The activation of the NLRP1 inflammasome can promote the secretion of high mobility group protein B1 (HMGB1), which stimulates inflammatory responses by modulating both the innate and the adaptive immune responses (54).

#### NLRC4

NLRC4 plays an important role in protection against certain Gram-negative bacteria with type III or type IV secretion systems (55). NLRC4 combines with NAIPs (NLR family apoptosis inhibitory proteins)to forms complexes and activates NLRC4 by binding to pathogenic proteins from flagellin or type III/IV secretion systems. NLRC4 oligomerizes and directly interacts with pro-Caspase 1 *via* a CARD-CARD interaction, bypassing the participation of ASC, when the NLRC4 is activated (55). There are several gain-of-function mutations in NLRC4, which have been shown to result in the development of autoinflammatory diseases (56).

#### NLRP6

NLRP6 causes ASC speck formation and subsequently Caspase1 activation (57). It has been reported that NLRP6 plays an inflammasome-dependent role in host defenses and inflammation and an inflammasome-independent role in intestinal homeostasis and cancer. However, the molecular mechanisms in these processes are not fully explained (58, 59).

## Role of Pyroptosis in Cardiac Remodeling

### Role of Pyroptosis in Cardiac Fibrosis

Cardiac fibrosis, the expansion of the cardiac interstitium due to a net accumulation of extracellular matrix (ECM) proteins, occurs primarily in cardiac fibroblasts and accompanies most cardiac pathologic conditions (60). Activated fibroblasts contribute to the regulation of matrix remodeling by producing proteases, such as matrix metalloproteinases (MMPs). During mild injury, extracellular matrix components are temporarily accumulated and quickly eliminated, promoting the recovery of normal tissue structure (61, 62), however, when damage is severe, extracellular matrix components continue to accumulate, leading to structural destruction, organ dysfunction, and ultimately organ failure (63). Cardiac fibrosis has a dual role, although, in most myocardial diseases, the extent of cardiac fibrosis predicts adverse outcomes, in myocardial infarction, reparative fibrosis performs an important repair function, preventing heart rupture (60). Cardiac fibrosis is an important manifestation of cardiac remodeling. It has been found in various cardiovascular diseases that cardiac fibrosis is closely related to pyroptosis, which has an important influence on the occurrence and development of HF.

In a mouse model of HF with transverse aortic constriction (TAC), the content of NLRP3 increased and the degree of myocardial fibrosis increased (64–66). Immunofluorescence was applied to determine the cellular localization of NLRP3 protein in cardiac tissue (67). NLRP3 inflammasome activation mainly occurs in cardiac fibroblasts during myocardial remodeling and repair (68), activates IL-1β release and pyroptosis in cardiac fibroblasts after myocardial infarction (MI) (69).A significant increase in expression of pyroptosis and MMP9 in cardiac fibrosis in diabetes (33, 70). Cardiac dysfunction, cardiac fibrosis (TGF-β1, collagen I, and collagen III), pyroptosis (Caspase-1, IL-1β, and GSDMD) were found in diabetic rats (71). Inhibition of Caspase-1 reduced the occurrence of cardiac fibrosis in diabetic cardiomyopathy and improved cardiac function *via* regulation of miR-135b (71). The proinflammatory cytokines released by pyroptosis activate fibroblasts and promote tissue fibrosis (63, 72). IL-1β and IL-18 promote Ca^2+^ efflux of the sarcoplasmic reticulum, induces myocardial interstitial fibrosis, activates TNF-α release, and TNF-α reacts on Caspase-1, forming an inflammatory cascade loop and promoting the progression of HF (73, 74).

Activation of the NLRP3 inflammasome was observed in LPS-stimulated cardiac fibroblasts and myofibroblasts, suggesting that the NLRP3 inflammasome and pyroptosis may contribute to myocardial dysfunction upon pyroptosis (16, 73).

### Role of Pyroptosis in Myocardial Hypertrophy

Myocardial hypertrophy is divided into physiological and pathological. Physiological cardiac hypertrophy is of great significance for maintaining cardiac efficiency, however, hypertrophic cardiomyopathy, long-term uncontrolled systolic hypertension, and continuous overload pressure from cardiac valve stenosis can promote cardiac remodeling and lead to HF (75, 76).

Factors related to pyroptosis play an important role in myocardial hypertrophy. There was a report that hyperactivated NLRP3 inflammasome with pyroptosis of cardiomyocytes were presented in the myocardial tissues of dilated cardiomyopathy patients, which were negatively correlated with cardiac function (77). *In vitro* and *in vivo* models of cardiac hypertrophy, the expression levels of pyroptosis-related factors were significantly increased (66), downregulation of Caspase-1 and IL-1β expression by Caspase-1 inhibitors attenuates angiotensin II (Ang II) -induced cardiac hypertrophy (78). A recent study indicated that NLRP3 levels in C57/BL6 mice with chronic pressure overload-induced by TAC were significantly increased and were involved in the production of inflammatory mediators and fibrosis factors, leading to myocardial fibrosis, myocardial hypertrophy, and impaired cardiac function (64). Under pressure overload, S-nitrosylation of muscle LIM protein (MLP) increased the complex formation between toll-like receptor 3 (TLR3) and receptor-interacting protein kinase 3 (RIP3), inducing NLRP3 inflammasome activation, and promoting the development of myocardial hypertrophy. Pharmacologic blockade or RNA interference of NLRP3 and inhibition of IL-1β can reduce pressure overload-induced myocardial hypertrophy (79). Meanwhile, IL-18 expression was significantly increased during pressure overload in rabbit models with TAC (80). There is good evidence that the heart weight/body weight ratio was significantly increased in the diabetic cardiomyopathy group compared to the control (33). Elmadbouh I and Singla DK found that diabetic cardiomyopathy involves sterile inflammation and causes the upregulation of NLRP3-Nek7-GBP5 inflammasome complex, which finally initiates Caspase-1-dependent pyroptosis in diabetic cardiomyopathy. Inflammation-induced pyroptosis has adverse effects on diabetic cardiac remodeling, endothelial progenitor cells, neovascularization, and cardiac function (33). Meanwhile, silica nanoparticles (SiNPs) exposure is correlated with adverse cardiovascular effects, literature suggested SiNPs could trigger pyroptosis and cardiac hypertrophy *via* ROS/NLRP3/Caspase-1 signaling pathway (81). miR-133A-3p could target IKKε to inhibit pyroptosis, alleviate myocardial hypertrophy, and protect cardiac function (82). In H9C2 cardiomyocytes, NF-κB, NLRP3, and receptor of advanced glycation endproducts (RAGE) induced hypertrophy through the RAGE-NF-κB- NLRP3- IL-1β signaling pathway (83).

### Role of Pyroptosis in Excessive Inflammation

Cardiomyocyte necrosis and inflammation play key roles in the pathophysiology of cardiovascular diseases. The inflammatory response is mainly to repair the heart, but the excessive inflammatory response will lead to cardiac dysfunction, adverse cardiac remodeling, and HF (14).

NLRP3 inflammasome contributes a lot to sterile inflammatory response and pyroptosis in ischemia/reperfusion (I/R) injury (67). Inflammation triggered by pyroptosis (NLRP3)-related pyroptosis of cardiac fibroblasts (CFs) resulted in cardiomyocytes death and myocardial dysfunction (67). It was found that NLRP3 and other inflammatory factors were generally elevated in myocardial cells after MI, and the subsequent response was mainly generated by the activation and release of inflammatory factors (84). Meanwhile, cell fragments and metabolites act as DAMPs to activate inflammasome and membrane P2X7 receptor channels, causing K^+^ efflux, activating NLRP3, the activated NLRP3 to recruit ASC and pro-Caspase-1, activates Caspase-1, Caspase-1 splines pro-IL-1 β, and other pro-inflammatory cytokines into active mature bodies that recruit and activate other immune cells and induce the synthesis of chemokines, inflammatory cytokines, and adhesion factors, further amplifying the inflammatory response (17, 85–88). Cardiomyocyte H/R induced the release of the inflammatory factor IL-18, which is associated with pyroptosis, in the cell culture supernatant, but there was no IL-1β release. IL-18 further amplifies the inflammatory cascade by inducing additional cytokines, adhesion molecules, and chemokines. Neutralization of IL-18 significantly attenuates I/R-induced tissue damage *in vivo*. Streptozotocin (STZ)-induced diabetic cardiomyopathy significantly increased inflammasome formation (TLR4, NLRP3, Nek7, and GBP5), which induced the occurrence of pyroptosis, accompanied by the increased of inflammatory cytokines (IL-6 and TNF-α), MMP9, infiltration of monocytes (CD14), macrophage (iNOS), and dendritic cells (CD11b and CD11c). Moreover, significant endothelial progenitor cells (EPCs) dysfunction (c-Kit/FLk-1, CD31), adverse cardiac remodeling, and reduction in left ventricular (LV) heart function were observed (33). BMP-7 reduced inflammation and improved adverse myocardial remodeling, hypertrophy as well as interstitial and vascular fibrosis (33).

The expression of IL-1β was mainly derived from fibroblasts and IL-1β was not significantly altered in cardiomyocytes under oxidative stress conditions, cardiomyocyte pyroptosis, and release of the proinflammatory cytokine IL-18 may activate cardiac fibroblasts to induce the secondary production of cytokines (89). Caspase-1 is an important regulator of the inflammatory response, activated Caspase-1 can trigger pyroptosis, and the release of pro-inflammatory cytokines IL-1β and IL-18 can cause the amplification of inflammatory cascade, resulting in endothelial dysfunction, and then produce or increase the development of myocardial fibrosis. ROS produced by oxidative stress can activate the NLRP3 inflammasome, leading to Caspase-1 activation and IL-1β secretion (90–92). When the ROS system of cardiomyocytes is activated, the accumulated ROS and Ca2^+^ are released into the cytoplasm, and the mitochondrial membrane potential changes, resulting in pyroptosis. Pirfenidone can regulate the ROS-dependent NLRP3-IL-1β signaling pathway by inhibiting NLRP3, improving left ventricular hypertrophy and myocardial fibrosis in rats with TAC (93).

### Role of Pyroptosis in Cardiomyocytes Death and Myocardial Dysfunction

Pyroptosis directly leads to the death of cardiomyocytes, which reduces the number of effective cardiomyocytes, thereby affecting the systolic and diastolic functions of the myocardium and promoting cardiac remodeling in HF. In adult mouse cardiomyocytes, the absence of GSDMD markedly blocked H/R-induced cardiomyocyte pyroptosis, which is associated with N-terminal fragment cleavage release (94, 95). Immunoblot analysis revealed significantly increased levels of GSDMD and GSDMD-N after H/R in adult mouse cardiomyocytes in a time-dependent manner, bring with large numbers of balloon-shaped vesicles and accumulation of propidium iodide (PI), which are typical characteristics of pyroptosis, as well as exhibited decreased ATP levels and the loss of cell membrane integrity. The mouse myocardial I/R injury model showed that GSDMD deficiency significantly reduced I/R-induced myocardial infarct size. Serum GSDMD levels were also significantly higher after percutaneous coronary intervention in patients with ST-segment–elevation myocardial infarction than in age-matched stable coronary artery disease patients (89). Moreover, trimetazidine and silencing PVT1 could alleviate myocardial I/R damage through suppressing GSDMD-mediated pyroptosis *in vivo* and in vitro, involved TLR4/MyD88/NF-κB/NLRP3 inflammasome pathway, improved cardiac fibrosis, inflammatory cytokines, and cardiac function (94, 96). NLRP3 markedly promotes pyroptosis in the progression of AMI, knockdown of NLRP3 attenuated cardiomyocyte pyroptosis and significantly decreased the infarct size, as evidenced by decreased expression levels of ASC, pro-Caspase-1, Caspase-1-p10, GSDMD, cleaved GSDMD, and IL-18 (45). Becn1-driven autophagy is a protective response in the heart during I/R, Becn1 overexpression suppressed Caspase-4 inflammasome activation and pyroptosis, alleviated microvascular damage, reduced infarct size, and mitigated cardiac inflammation and cell death (97). The recent research showed that activation of SIRT1 by inhibition of miR-29a inhibited oxidative stress, pyroptosis and protect I/R injury (98, 99). It has been found that pyroptosis participated in the pathogenesis of sepsis-induced myocardial injury which was associated with the XIST/miR-150-5p/c-Fos axis and ER/SIRT1/NLRP3/GSDMD pathway (99, 100).

Echocardiogram data suggests impaired LV function in diabetic cardiomyopathy, diabetic cardiac systolic and diastolic dysfunction can be preserved by inhibiting pyroptosis proteins (33), CD74 ablation protects against Type 2 diabetes-induced cardiac remodeling and contractile dysfunction through NLRP3/pyroptosis-mediated regulation of ferroptosis (101). IL-1β released by pyroptosis stimulates the synthesis of inducible nitric oxide synthase (iNOS), leading to cell death and cardiac remodeling leading to HF (74, 102). In the progression of HF, the activation of the NLRP3 inflammasome leads to the release of IL-1β, which can induce Ca2^+^ efflux in the myocardial plasma reticulum, directly affecting the excitation-contraction coupling of the myocardium, and impairing the systolic function of the myocardium (74). It was found that the amount of ASC, NLRP3, and Caspase-1 in myocardial cells at the edge of AMI increased significantly with time, and the level of NLRP3 in myocardial fibroblasts in ischemic myocardium increased significantly, inhibition of NLRP3 activation could reduce the size of MI and preserve the function of myocardial after infarction (85).

## Potential Clinical Applications Of Pyroptosis to Ameliorate Adverse Cardiac Remodeling in HF

The improvement of cardiac remodeling plays an important role in maintaining cardiac function and improving patients' quality of life. At present, there are many inhibitors targeting factors related to the pyroptosis pathway, and these studies provide a feasible way to better solve the problem of pyroptosis to improve cardiac remodeling in HF. Although many inhibitors of pyroptosis-related molecules have been studied (20, 103, 104), there is still a certain gap between them and clinical use. For this reason, we reviewed some common clinical drugs and effective ingredients of natural drugs targeting pyroptosis and put forward some possible targeting molecules to provide readers with certain ideas.

### Therapy for Improving Cardiac Remodeling by Drugs on Pyroptosis

MCC950 inhibited inflammation in early myocardial infarction, reduced cardiac fibrosis, and protected cardiac function (105), combined with Rosuvastatin (RVS), MCC950 inhibited the expression of NLRP3, Caspase-1, interleukin-1β, and Gasdermin D n-terminal domains, and decreased serum lactate dehydrogenase (LDH) level, improved cardiac systolic function and myocardial fibrosis in mice (106). Metformin inhibited the activation of TNF-α, IL-6, IL-1β, and NLRP3 inflammasome, reduced the size of MI and myocardial fibrosis, enhanced the activity of myocardial cells, reduced the activity of LDH, inhibited pyroptosis and inflammation (107). Trimetazidine (TMZ) and Emodin increased the viability of H9c2 cardiomyocytes subjected to H/R treatment and reduced the infarct size *in vivo* as well as alleviated pyroptosis induced by myocardial I/R injury through the TLR4/MyD88/NF-κB/NLRP3 inflammasome pathway. Therefore, TMZ represents an alternative treatment for myocardial I/R injury (94, 108). Liraglutide alleviated pyroptosis mediated by NLRP3 inflammasome by down-regulating the SIRT1/NOX4/ROS pathway in H9C2 cells (109). Artemisinin and Triptolide have protective effects on myocardial function, which is related to the reduction of factors involved in pyroptosis (64, 110). It was shown that pinocembrin can inhibit pyroptosis by activation of Nrf2/Sirt3 signaling pathway, LVEF, LVFS, LVIDd, LVID, and myocardial fibrosis were improved, and the expressions of LDH, CK-MB, IL-1β, and IL-18 were reduced (111). Pyrroloquinoline quinone inhibited ROS and NF-κB activation inhibited NLRP3 inflammasome and Caspase-1, IL-1β and IL-18 expression, and improved myocardial hypertrophy and cardiac fibrosis (112). Ranolazine treatment of diabetic cardiac fibrosis inhibited pyroptosis and collagen deposition by upregulating miR-135b (71). Syringaresinol (SYR) improved cardiac function and alleviated myocardial injury in sepsis-induced cardiac dysfunction mouse *via* the estrogen receptor (ER)/SIRT1/NLRP3/GSDMD pathway (99). Irisin protected cardiac function by inhibiting NLRP3 and ameliorating cardiomyocyte hypertrophy induced by pyroptosis (66). Piperazine ferulate can suppress the I/R-triggered NLRP3 inflammasome activation and pyroptosis (113). Sevoflurane decreased heart-type fatty acid-binding protein (H-FABP), ischemia modified albumin (IMA), IL-1β, and IL-18 in serum, and alleviated myocardial injury in patients with myocardial ischemia (114). Sevoflurane reduced the H/R rats' injury of cardiomyocytes and protected the cardiac function by regulating inflammatory reaction and pyroptosis by inhibiting the P2X7-NLRP3 signaling pathway (114). These drugs interfere with pyroptois and improve cardiac remodeling from various mechanisms, which has certain practical significance (Table 1).

**Table 1:** Therapy for improving cardiac remodeling by drugs on pyroptosis.

**Drugs**	**Models**	**Mechanism**	**Effects**	**Ref**
Rosuvastatin MCC950	CME rats	Rosuvastatin decreased the expression of NLRP3, Caspase-1, IL-1β, and GSDMD N-terminal domains, which is associated with regulating mitochondrial ROS	Pyroptosis (NLRP3, Caspase-1, IL-1β, GSDMD)↓; Cardiac fibrosis↓ Cardiac systolic function↑; Cardiac remodeling↓	(105, 106)
Metformin	I/R	Metformin protects against myocardial ischemia-reperfusion injury and cell pyroptosis *via* AMPK/NLRP3 inflammasome pathway	Pyroptosis (NLRP3, IL-1β)↓; Cardiac fibrosis↓; Inflammation (TNF-α, IL-6)↓; Myocardial infarct size↓, Cardiomyocyte activity (LDH↓)↑	(107)
Trimetazidine/ Emodin	I/R	Trimetazidine/ Emodin alleviated pyroptosis induced by myocardial I/R injury through the TLR4/MyD88/NF-κB/NLRP3 inflammasome pathway.	TLR4, MyD88, phospho-NF-κB p65, the NLRP3 inflammasome↓; Infarct size↓Viability of H9c2 cardiomyocytes↑	(94, 108)
Liraglutide	Hypoxia H9C2	Liraglutide alleviated pyroptosis mediated by NLRP3 inflammasome by down-regulating the SIRT1/NOX4/ROS pathway	Pyroptosis (NLRP3, Caspase-1 p20, GSDMD-N)↓; Cardiomyocyte activity (LDH↓)↑	(109)
Pinocembrin	DOX-induced cardiotoxicity	Pinocembrin inhibited DOX-induced cardiomyocyte pyroptosis *via* activating Nrf2/Sirt3 signal pathway.	Pyroptosis (IL-1β, IL-18)↓; Cardiac fibrosis↓; Cardiac function (LVEF, LVFS, LDH, CK-MB)↑	(111)
Pyrroloquinoline quinone	DCM	Pyrroloquinoline quinone improved DCM in diabetic mice by inhibiting NF-κB/NLRP3 inflammasome-mediated cell pyroptosis.	Pyroptosis (NLRP3, Caspase-1, IL-1β, IL-18)↓; Cardiac fibrosis (collagen I and TGF-β1)↓; Myocardial hypertrophy (ANP and BNP)↓	(112)
Artemisinin	I/R	Artemisinin inhibited cardiac autophagy, improved mitochondrial electron transport chain activity, decreased activation of NLRP3 inflammasome.	Pyroptosis (NLRP3, ASC, cleaved Caspase-1, IL-1β)↓Infarct size and CK-MB, LDH↓ Cardiac autophagy↓	(110)
Ranolazine	DCM	miR-135b directly bound to Caspase-1	Pyroptosis (Caspase-1, IL-1β, GSDMD)↓; Cardiac Fibrosis (TGF-β1, collagen I and collagen III)↓; Cardiac function↑	(71)
Sevoflurane	I/R patients with myocardial ischemia	Sevoflurane inhibited the expression of IL-1β, IL-18, and GSDMD by inhibiting the P2X7-NLRP3 signaling pathway	Pyroptosis (NLRP3, Casepase-1, GSDMD, IL- 1β, IL-18)↓; Cardiac injury (CK, CK-MB, LDH, MDA, SOD)↓; Inflammation (CD11b)↓	(114)
Piperazine ferulate	I/R	Piperazine ferulate can suppress the I/R-triggered NLRP3 inflammasome activation and pyroptosis	Pyroptosis (NLRP3, Caspase-1, GSDMD, IL- 1β, ASC)↓ Cardiac function (LVEF, LVFS↑, mitral early diastolic flow velocity/late diastolic flow velocity, infarction size↓)↑	(113)
Iguratimod	I/R	Iguratimod protected cardiomyocytes by reducing the cascade of inflammation in the heart by inhibiting cardiac fibroblast pyroptosis *via* the COX2/NLRP3 signaling pathway.	Pyroptosis (NLRP3, Casepase-1, GSDMD, IL- 1β, IL-18)↓; Inflammatory response (IL-6, TNF-α)↓	(67)
Syringaresinol	Sepsis mouse	Syringaresinol ameliorated sepsis-induced cardiac dysfunction *via* the ER/SIRT1/NLRP3/GSDMD pathway.	Proinflammatory cytokines↓; Cardiac function↑	(99)

*CME, coronary microembolization; I/R, myocardial reperfusion; ROS, reactive oxygen species; LDH, lactate dehydrogenase; TNF-α, tumor necrosis factor α; NF-κB, nuclear-factor-κB; TLR4, toll-like receptors 4; SIRT1, Sirtuin 1; NOX4, NADPH oxidase 4; Dox, doxorubicin; CK-MB, creatine kinase-MB; DCM, diabetic cardiomyopathy; cTnI, cardiac troponin I; EF, ejection fraction; FS, fractional shortening; CO, cardiac output; SV, stroke volume; ANP, atrial natriuretic peptide; BNP, brain natriuretic peptide; MDA, malondialdehyde; SOD, superoxide dismutase; ER, estrogen receptor*.

### Therapy for Improving Cardiac Remodeling by Potential Molecules on pyroptosis

Mixed lineage kinase 3 (MLK3) mainly regulates NF-κB/NLRP3 signaling pathway-mediated inflammation and that pyroptosis causes myocardial fibrosis in the early stages of CHF (65). Knockdown of retinol-binding protein 4 (RBP4) in heart attenuates cardiac pyroptosis in AMI *via* interaction with NLRP3, hypertrophic markers (atrial natriuretic peptide, brain natriuretic peptide, and myosin heavy chain 7)were decreased in the left ventricular myocardium of RBP4 knockdown mouse, and echocardiography demonstrated that the left ventricular internal dimension and left ventricular volume were also decreased by inhibition of RBP4, indicating attenuated adverse cardiac remodeling. Importantly, knockdown of RBP4 significantly improved AMI-induced decrease of left ventricular ejection fraction and fractional shortening (45). Meanwhile, bone morphogenetic protein-7 (BMP-7)can inhibit the NLRP3 inflammasome complex and their activator Nek7-GBP5, and the subsequent cascade of pyroptosis in diabetic cardiomyopathy, attenuate inflammatory infiltrated dendritic and M1 macrophages, reducing inflammation and adverse cardiac remodeling through enhanced neovascularization following BMP-7 treatment while improving heart function BMP-7 attenuated inflammation-induced pyroptosis, adverse cardiac remodeling, and improved heart function *via* the TLR4-NLRP3 inflammasome complex activated by novel signaling Nek7/GBP5 (33). Becn1 overexpression increased Ischemia-reperfusion mouse survival and decreased the levels of serum LDH and CK. BECN1 attenuated F4/80+ macrophages and CD11b+ neutrophils infiltration in the heart (97). Meanwhile, myocardial fibrosis was markedly ameliorated, the collagen was decreased through suppressing GSDMD-mediated pyroptosis when PVT1 knockdown (96). miR-135b can alleviate the fibrosis of cardiac fibroblasts, as well as cardiac fibroblast pyroptosis, which can be inhibited *via* miR-135b that is directly bound to Caspase-1 (71). LncRNA KLF3-AS1 in exosomes secreted from hMSCs by acting as a ceRNA to sponge miR-138-5p can regulate Sirt1 to inhibit pyroptosis and attenuate MI progression (84). Interferon regulatory factor 2 (IRF2) is directly bound to the GSDMD promoter to drive GSDMD transcription and promote pyroptosis and IRF2 expression may be regulated *via* the HIF-1 signaling pathway (95). Aldehyde dehydrogenase 2 (ALDH2) has been proven to protect the heart and brain against regional I/R injury, in which the protective role is related to the inhibition of pyroptosis. ALDH2 activator N- (1,3-benzodioxol-5-ylmethyl)-2,6-dichloro-benzamide (Alda-1) would improve post-resuscitation cardiac and neurological outcomes in a clinically relevant swine model of cardiac arrest and resuscitation (115). Silencing CMKLR1 could inhibit the expression of activated Caspase-1 and IL-1β, and reduce the occurrence of pyroptosis (116). Resolvin D2 (RvD2), an innate inflammatory suppressor produced by ω3 polyunsaturated fatty acids, has been found to promote NLRP3 degradation through autophagy. IL-1β secretion is reduced in the presence of exogenous RvD2 *in vivo* and *in vitro*, which may be a potential therapeutic target for inflammasome (117). There are reports that soluble receptors for advanced glycation end-products (sRAGE) would not only improve cardiac function and diminish the infarction size but also reduce the occurrence of apoptosis, necrosis, and pyroptosis in I/R-treated myocardium. Meanwhile, sRAGE also reduced the levels of pyroptosis-related proteins in cardiomyocytes, such as NLRP3, GSDMD-NT, IL-1β, and IL-18, which were related to the NF-κB pathway (118). These molecules can improve cardiac remodeling by targeting pyroptosis in various pathways or directly, which has certain research value (Table 2).

**Table 2:** Therapy for improving cardiac remodeling by potential molecules on pyroptosis.

**Potential molecules**	**Models**	**Mechanism**	**Effects**	**Refs**
MLK3 miR-351	TAC	MLK3 mainly regulates NF-κB/NLRP3 signaling pathway-mediated inflammation and that pyroptosis causes myocardial fibrosis in the early stages of CHF	Pyroptosis↓; Cardiac hypertrophy↓; Cardiac Fibrosis↓; Cardiac function↑	(65)
LncRNA PVT1	H/R-treated H9C2 cells	Silencing PVT1 could alleviate myocardial I/R damage by suppressing GSDMD-mediated pyroptosis	Pyroptosis (GSDMD-N↓)↓; Cardiac Fibrosis↓; Inflammatory cytokines↓; Cardiac function (α-MHC↑and β-MHC↓)↑	(96)
NOX1, NOX4, Drp1	DCM	Dox enhanced expressions of NOX1 and NOX4 and induced mitochondrial fission through dynamin-related protein 1 activation, leading to NLRP3 inflammasome-mediated pyroptosis in cardiomyocytes *via* Caspase-1-dependent manner.	Pyroptosis (NLRP3, ASC, Caspase-1, IL-1β, IL-18,)↓	(77)
BMP-7	DCM	BMP-7 activated the TLR4-NLRP3 inflammasome complex by signaling Nek7/GBP5.	Pyroptosis (Caspase-1, IL-1β, IL-18,)↓; Cardiac fibrosis (MMP-9) ↓; Cardiac hypertrophy and dilation↓; Inflammasome formation (TLR4-NLRP3)↓; Inflammatory cytokines (IL-6, TNF-α)↓; Inflammatory cells (CD14, iNOS, CD11b, CD11c)↓; Adverse cardiac remodeling↓; EPC markers and neovascularization (c-Kit/Flk-1 and CD31/α-SM actin)↑; Cardiac function↑	(33)
Becn1	I/R	Becn1 overexpression suppressed Caspase-4 inflammasome activation and pyroptosis by enhancing autophagic flux.	Pyroptosis (Caspase-4, IL-1β, GSDMD) ↓; Inflammation (F4/80+ macrophages and CD11b+ neutrophils infiltration in the heart)↓; Autophagic flux (Beclin1, LC3-II/LC3I)↑; Myocardial infarct size (LDH, CK↓)↓	(97)
sRAGE	I/R	sRAGE protected the heart from pyroptosis by inhibiting the NF-κB pathway during myocardial ischemia-reperfusion.	Pyroptosis (NLRP3, Casepase-1, GSDMD, IL- 1β, IL-18) ↓; Cardiac function (the movement of the left ventricle anterior wall, CO, SV, EF, FS)↑Myocardial infarct size (cTnI)↓	(118)
METTL3	I/R	METTL3 promoted DGCR8 binding to pri-miR-143-3p through m^6^A modification, thus enhancing miR-143-3p expression to inhibit PRKCE transcription and further aggravating cardiomyocyte pyroptosis and MI/R injury.	Pyroptosis (NLRP3, Casepase-1, GSDMD-N, IL- 1β, IL-18)↓Myocardial injury↓	(119)
RBP4	AMI	RBP4 interacted directly with NLRP3 in cardiomyocytes, promoted the precursor cleavage of Caspase-1, and subsequently induced GSDMD dependent pyroptosis.	Pyroptosis (GSDMD, ASC, pro-Caspase-1, Caspase-1-p10, GSDMD, cleaved GSDMD, and IL-18) ↓; Hypertrophic markers (ANP, BNP, and MHC7) ↓; Myocardial infarct size↓; Adverse cardiac remodeling (left ventricular internal dimension and left ventricular volume) ↓Cardiac function (EF, FS)↑	(45)
IRF2	MI	IRF2 is directly bound to the GSDMD promoter to drive GSDMD transcription and promote pyroptosis and IRF2 expression may be regulated *via* the HIF-1 signaling pathway.	Pyroptosis (Cleaved caspase-1, IL-1β, IL-18, GSDMD-N, GSDMD) ↓; Cardiac function (EF, FS)↑	(95)
LncRNA KLF3-AS1	MI	LncRNA KLF3-AS1 in exosomes secreted from hMSCs by acting as a ceRNA to sponge miR-138-5p can regulate Sirt1 to inhibit pyroptosis and attenuate MI progression.	Apoptosis and pyroptosis↓; Myocardial infarct size↓	(84)
miR-762	I/R	Delivery of exogenous miRNA-762 before transplantation significantly increased the post-transplant survival of stem cells and also significantly ameliorated cardiac fibrosis and heart functions following I/R injury.	Pyroptosis (Caspase-1, Caspase-11, Caspase-1, GSDMD, IL-1β)↓; Cardiac function↑	(120)

*TAC, transverse aortic constriction; MLK3, mixed lineage kinase 3; I/R, myocardial reperfusion; H/R, hypoxia/reoxygenation; MHC, myosin heavy chain; RBP4, retinol-binding protein 4; BMP-7, bone morphogenetic protein-7; MMP-9, matrix-metalloprotianse-9; TNF-α, tumor necrosis factor α; TLR4, toll-like receptor-4; sRAGE, soluble receptor for advanced glycation end-products; cTnI, cardiac troponin I; EF, ejection fraction; FS, fractional shortening; CO, cardiac output; SV, stroke volume; ANP, atrial natriuretic peptide; BNP, brain natriuretic peptide; IRF2, Interferon regulatory factor 2; HIF-1, hypoxia inducible factor 1; ER, estrogen receptor; METTL3, methyltransferase-like protein 3; PRKCE, protein kinase C epsilon; Dox, doxorubicin; NOX, oxidase*.

Interestingly, hydrogen improved cardiac function and reduced the area of cardiac fibrosis by inhibiting NLRP3-mediated pyroptosis, and it has been demonstrated in vitro that hydrogen alleviated cardiomyocyte damage induced by hypoxia and myocardial fibroblast migration and activation induced by Ang II (121). As an important regulator of IL-1β production and subsequent pyroptosis, delivery of exogenous miRNA-762 before transplantation significantly increased the post-transplant survival of stem cells and also significantly ameliorated cardiac fibrosis and heart functions following I/R injury (120). Mesenchymal stem cells (MSCs), derived from bone marrow, placenta, adipose, or other tissues, significantly alleviated cardiac arrest cardiac injuries in swine, in which the protective effects were related to the inhibition of cell pyroptosis and ferroptosis (122).

## Concluding Remarks and Perspectives

**Figure 2:**
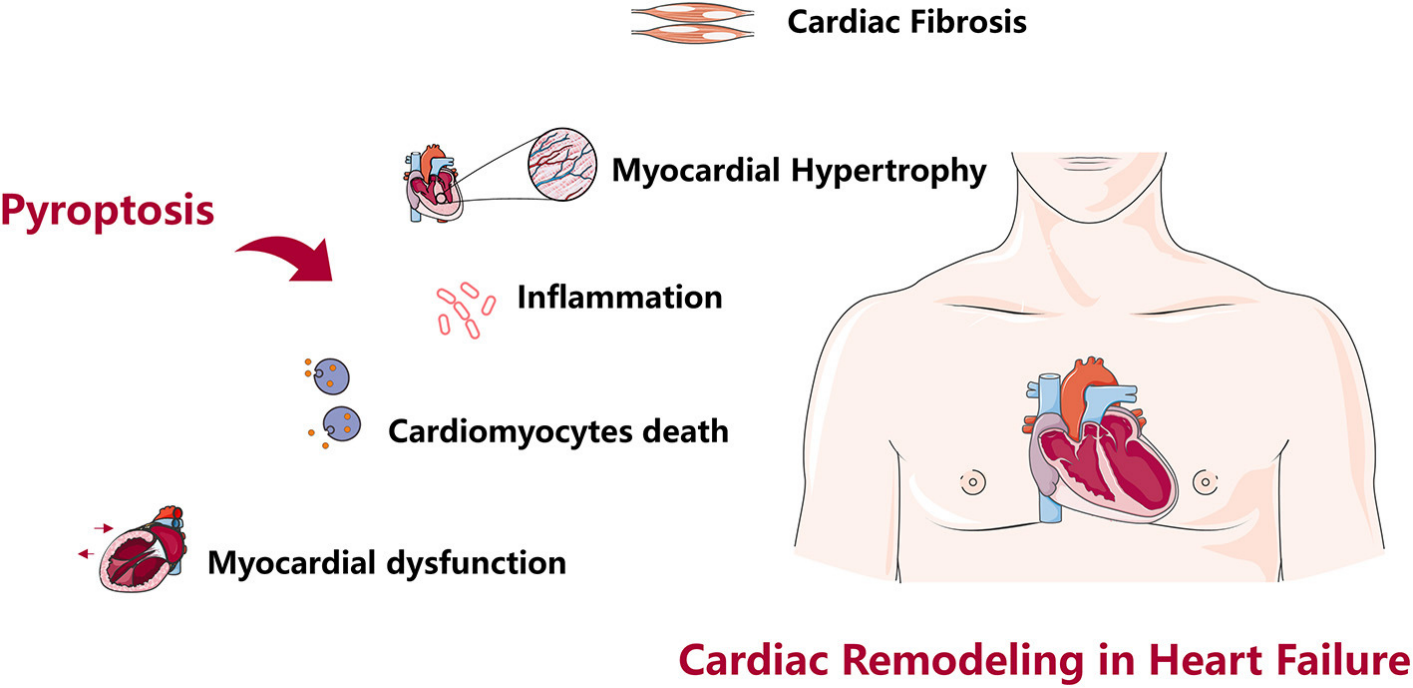
Role of pyroptosis in cardiac remodeling. Pyroptosis causes cardiac fibrosis, cardiac hypertrophy, cardiomyocytes death, myocardial dysfunction, excessive inflammation, and cardiac remodeling in heart failure.

Adverse cardiac remodeling is a decisive factor in the progression of clinical HF, pyroptosis is involved in various stages of cardiac fibrosis, cardiac hypertrophy, cardiomyocytes death, myocardial dysfunction, and excessive inflammation, and these factors often overlap to promote cardiac remodeling in HF (123) (Figure 2) Many targeted inhibitors have been developed for pyroptosis, and there are also relevant clinical drugs that can inhibit pyroptosis and improve cardiac remodeling, however, it is undeniable that studies focus on NLRP3 and Caspase-1 inhibition, while there are few studies on another inflammasome (27, 72). Research on improving cardiac remodeling in HF from the perspective of pyroptosis has a good prospect, however, current studies mostly focus on improving the protein indexes and pathological observation related to pyroptosis and cardiac remodeling, and lack of in-depth molecular interaction mechanism, and the theoretical mechanism is not clear, so it is difficult to achieve clinical transformation of research results. In-depth discussion of the molecular interaction mechanism and targeting another inflammasome to improve cardiac remodeling will become our focus in the future.

## Author Contributions

RC, WX, and SS conceived and designed the study. YZ and YL involved in a database search extracted the data. QS, YH, and YL analyzed the data and wrote the manuscript. YH polished the English. HW and YH revised the manuscript. All authors listed approved it for publication.

## Funding

This work was supported by the National Natural Science Foundation of China (Grant Nos. 81904189 and 82074409).

## Conflict of Interest

The authors declare that the research was conducted in the absence of any commercial or financial relationships that could be construed as a potential conflict of interest.

## Publisher's Note

All claims expressed in this article are solely those of the authors and do not necessarily represent those of their affiliated organizations, or those of the publisher, the editors and the reviewers. Any product that may be evaluated in this article, or claim that may be made by its manufacturer, is not guaranteed or endorsed by the publisher.
